# Delivery of Anti-miRNA-221 for Colorectal Carcinoma Therapy Using Modified Cord Blood Mesenchymal Stem Cells-Derived Exosomes

**DOI:** 10.3389/fmolb.2021.743013

**Published:** 2021-09-20

**Authors:** Siqi Han, Guangchao Li, Meng Jia, Yulu Zhao, Chenglong He, Mengxi Huang, Longwei Jiang, Meijuan Wu, Jiahe Yang, Xiaoqin Ji, Xiaobei Liu, Cheng Chen, Xiaoyuan Chu

**Affiliations:** ^1^ Department of Medical Oncology, Jinling Hospital, Nanjing, The First School of Clinical Medicine, Southern Medical University, Guangzhou, China; ^2^ Department of Medical Oncology, Jinling Hospital, School of Medicine, Nanjing University, Nanjing, China; ^3^ Department of Medical Oncology, Jinling Hospital, Nanjing Medical University, Nanjing, China; ^4^ Department of Hematology, Guangdong Second Provincial General Hospital, Guangzhou, China; ^5^ School of Chemistry and Molecular Biosciences, University of Queensland, Brisbane, QLD, Australia

**Keywords:** anti-miRNA oligonucleotides, anti-miRNA-221, iRGD nanoparticles, delivery platform, exosomes, human cord blood mesenchymal stromal cells

## Abstract

**Background:** Exosomes, as natural intercellular information carriers, have great potential in the field of drug delivery. Many studies have focused on modifying exosome surface proteins to allow drugs to specifically target cancer cells.

**Methods:** In this study, human cord blood mesenchymal stromal cell-derived exosomes were used in the delivery of anti-miRNA oligonucleotides so as to be specifically ingested by tumor cells to perform anti-tumor functions. Mesenchymal stem cells modified by the fusion gene iRGD-Lamp2b were constructed to separate and purify exosomes, and the anti-miRNA-221 oligonucleotide (AMO) was loaded into the exosomes by electroporation.

**Results:** The AMO-loaded exosomes (AMO-Exos) effectively inhibited the proliferation and clonal formation of colon cancer cells *in vitro*, and it was further found that AMO-Exos was taken up by tumor cells through interaction with the NRP-1 protein. The results of a xenograft tumor model also showed that iRGD-modified exosomes were obviously enriched in tumor sites, exerting excellent anti-tumor efficacy. *In vivo* imaging showed that exosomes were mainly distributed in liver, spleen, and lung tissues.

**Conclusion:** Our results suggest that genetically modified exosomes could be an ideal natural nanostructure for anti-miRNA oligonucleotide delivery.

## Introduction

Anti-microRNA oligonucleotides (AMOs), or anti-miRNAs, bind miRNAs through complementary sequences and inhibit miRNA functions in cancer cells (Rupaimoole and Slack, 2017). The development of these drugs is based on in-depth descriptions of the biological pathogenesis between target miRNA and diseases (Li and Rana, 2014). Many preclinical studies have been performed on miRNA or anti-miRNA therapy since the first AMO drug, miravirsen (a specific inhibitor of miR-122), and entered clinical trials (Elmen et al., 2008). However, the biggest challenge to developing miRNA therapeutics is designing a miRNA delivery vector to prevent the degradation of nuclease and the escape of drug molecules from endocytosis (Xie et al., 2018). Furthermore, the dilemma of a miRNA drug delivery system lies in its potential immune stimulation and the lack of target specificity for the lesion (Taniguchi et al., 2019).

The homing ability of mesenchymal stem cells (MSCs) endows them with global positioning system navigation. It has been reported that MSCs using oncolytic herpes simplex virus can effectively kill malignant glioblastoma cells and prolong the median survival time (Cheng and Slack, 2012). MSC-derived exosomes have broad application possibilities because of their small size, low complexity, simple production, and easy storage (Zhao et al., 2019). It is an ideal delivery vector that can protect enzymes or RNA from degradation by wrapping molecules in a membrane and promoting intracellular uptake by endocytosis. In addition, exosomes are simple to transport in blood and easily pass through the blood-brain barrier. It was found that anti-miRNA-9 MSC exosomes can reverse the expression of multidrug transporters in resistant glioblastoma and reverse chemoresistance (Munoz et al., 2013).

The iRGD peptide is a specific peptide composed of nine amino acid residues (Yan et al., 2016). As a ligand, the iRGD peptide can interact with tumor cells with high expression of the neuropilin-1 (NRP-1) receptor, mediate the cell membrane penetration effect, and effectively kill the tumor (Nie et al., 2014). MicroRNA is a potential therapeutic target for many solid tumors. A significant number of studies have shown that miR-221 cancer-related miRNA, which is upregulated in colorectal cancer, liver cancer, lung cancer, and other malignant tumors (Park et al., 2011; Sun et al., 2011). MiR-221 downregulates key tumor suppressors, such as p27kip1, PTEN, and TIMP3, and has a significant effect on the cell cycle, apoptosis, and the Wnt signaling pathway (Fornari et al., 2008; Garofalo et al., 2009; Howe et al., 2012). The development of anti-miR221 has great significance in the treatment of solid tumors. In preclinical studies, however, it is difficult to deliver miRNA-related drugs efficiently to tumor cells, which hinders their broader application.

In this study, we loaded anti-miR221 into the exosomes of human cord blood MSCs (cbMSCs), which expressed the iRGD peptide. The anti-miR221 encapsulated in exosomes targeted NRP-1 receptor-positive tumor cells and might therefore play important roles in clinical application.

## Materials and Methods

### Cell Cultures

Human embryonic kidney cells (HEK293T) were obtained from Clontech (Mountain View, CA, United States). The human colon cancer cell lines Caco2 and HCT116 were obtained from the American Type Culture Collection. All cells were maintained in Dulbecco’s Modified Eagle Medium (DMEM) supplemented with 10% fetal bovine serum (FBS, Gibco), penicillin (100 U/mL, Gibco), and streptomycin (0.1 mg/ml, Gibco). The cells were cultured under standard conditions of 5% CO_2_ at 37°C. Immortalized cbMSCs (T0016; cbMSC-hTERT) were obtained from Applied Biological Materials Inc. (abm, Richmond, BC, Canada), and were maintained in Prigrow III medium (Cat. No. TM003; abm) containing 20% FBS and 4 ng/ml recombinant basic fibroblast growth factor at 37°C in a 5% CO_2_ incubator. The liquid was changed every 2–3 days after the suspension cells were discarded.

### Flow Cytometry

MSCsurface antigen markers were detected by flow cytometry. Briefly, 1 × 105 cells were washed and resuspended in 200 μL phosphate buffered saline (PBS). Then, the cell suspension was added with fluorescein isothiocyanate (FITC)-conjugated anti-CD73, allophycocyanin (APC)-conjugated anti-CD105, phycoerythrin-conjugated anti-CD34, and APC-conjugated anti-CD45 and incubated in the dark for 15 min. Subsequently, the cell suspension was washed with 2 ml PBS and centrifuged at 1000rpm for 5 min to remove the supernatant. Lastly, the cell suspension was added with 200 μL buffer solution and detected by a CytoFLEX flow cytometer (Beckman Coulter, United States). The expression of NRP-1 on 293T, Caco2, and HCT116 cells were detected using FITC-conjugated anti-human CD304 (neuropilin-1) antibody (Clone 12C2, Biolegend).

### Transfection and Lentiviral Transduction

The iRGD-Lamp2b fusion gene encodes (N to C) the signal peptide of Lamp2b (1-28aa), GNSTM glycosylation motif (GNSTM), 3 residue spacer (GSG), iRGD peptide (CRGDKGPDC), 10 residue spacer (GSGSGSGGSS), and Lamp2b (exosomal transmembrane protein, 29-410aa). The iRGD-Lamp2b fusion gene was constructed into lentivirus vector pLVX-IRES-Puro and transfected into 293T cells together with packaging plasmids psPAX2 and pMD2G. The cell supernatant was collected and the lentivirus particles (LV-iRGD-Lamp2b) were purified. cbMSCs were then infected by LV-iRGD-Lamp2b: 1 × 10^5^ cbMSCs cells were spread into 24-well plates 18 h before transfection. The next day, the original culture medium was replaced with fresh medium containing 6 μg/ml polybrene and LV-iRGD-Lamp2b with a multiplicity of infection (MOI) of 5. cbMSCs expressing the iRGD-Lamp2b fusion gene (MSC-iRGD) were obtained after continuous culture with 5 μg/ml puromycin (Sigma-Aldrich, United States).

### Isolation and Characterization of Exosomes

cbMSCs and cbMSC-iRGD cells at were cultured in DMEM with 10% FBS without exosomes. The supernatant of the cell culture (500 ml) was collected and centrifuged at low speed at 300×g for 10 min, then the supernatant was centrifuged at 2000×g for 10 min to remove the dead cells. The remaining supernatant was obtained and centrifuged at 10,000×g for 30 min to remove the cell debris. After that, the supernatant was centrifuged at high speed, 100,000×g for 70 min, and the crude exosome precipitation (containing a small amount of heteroproteins) was obtained. Lastly, the exosomes were resuspended with PBS and centrifuged at 100,000×g for 70 min to obtain pure exosomes. The exosomes’ morphology was identified by transmission electron microscopy (FEI Tecnai G2 Spirit Bio TWIN).

### Loading miRNAs Into Exosomes Using Electroporation

We employed AMOs with the following sequences: 5′GAA​ACC​CAG​ACA​GAC​AAU​GUA​GCU′3, with 2′ O-methyl modification. AMOs or FAM-labeled-miR-221 inhibitor (FAM-AMO) was loaded onto exosomes using electroporation methods. Briefly, 200 μg exosomes and 10 μL AMO were pre-mixed and added into the perforation dish. After electroporation (100 V, 125 μF, 1 ms), the exosome samples were incubated in a cell incubator for 1 h to promote exosome membrane repair. Subsequently, the mixed exosome samples were centrifuged twice at 100,000×g for 70 min each, and the supernatant was discarded. The sediment contained the compound exosomes loaded with miR-221 inhibitor (AMO-Exos), and stored at −80°C for standby use.

### Exosome Uptake

Various concentrations (0, 0.625, 1.25, 2.5, 5, 10, 20, 40 μg) of exosomes containing FAM-labeled-anti-miRNA-221 (iRGD-Exo^FAM−221^) were added into 293T, Caco2, and HCT116 cells. After culture for 24 h, FAM fluorescence was detected by flow cytometry after washing with PBS twice to assess the uptake efficiency of exosomes by tumor cells. The green FAM fluorescence was observed and photographed under an inverted fluorescence microscope (MF52-N; MSHOT, Guangzhou, China).

### Cell Proliferation Assay

We evaluated cell proliferation with a CellTiter 96® AQueous One Solution Cell Proliferation Assay (MTS) kit (Promega; G1112). We inoculated 3,000 Caco2 and HCT116 cells into flat 96-well plates. After overnight culture, various concentrations of AMO-Exos and their controls (AMO-NCs) were added. We continued to culture them for 24 h, with a small amount of CellTiter 96® AQueous single solution reagent added to the culture pores. After incubation for 4 h, the absorbance at 490 nm was recorded on a microplate reader. The absorbance value measured at 490 nm is directly proportional to the number of living cells in the culture.

### Quantitative Real-Time Polymerase Chain Reaction

Total RNA was extracted using the RNeasy Plus Universal Mini Kit (73,404, QIAGEN, Duesseldorf, Germany) or the miRNeasy Mini Kit (217,004, QIAGEN). Reverse transcription was performed to obtain complementary DNA with the QuantiTect Reverse Transcription Kit (205,311, QIAGEN). Real-time quantitative polymerase chain reaction (PCR) was performed using ABI7500 (7500, ABI, United States). The mRNA and miRNA expression data were normalized to glyceraldehyde 3-phosphate dehydrogenase (GAPDH) and U6, respectively. The primers used were as follows: miR-221-forward: 5ʹ-CCT GAA ACC CAG CAG ACA A-3ʹ, backward: 5ʹ-CAG GTC TGG GGC ATG AAC-3ʹ. U6-forward: 5ʹ-CTC GCT TCG GCA GCA CA-3ʹ, backward: 5ʹ-AAC GCT TCA CGA ATT TGC GT-3ʹ. NRP-1-forward: 5ʹ-GGC GCT TTT CGC AAC GAT AAA-3ʹ, backward: 5ʹ-TCG CAT TTT TCA CTT GGG TGA T-3ʹ. GAPDH-forward: 5ʹ-GGA GCG AGA TCC CTC CAA AAT-3ʹ, backward: 5ʹ-GGC TGT TGT CAT ACT TCT CAT GG-3ʹ.

### Colony Formation Assay

A total of 700 cells were pre-inoculated in a 6-well plate. After overnight culture, 20 μg exosomes containing AMO-Exos or AMO-NCs were added to Caco2 and HCT116 cells for another 14 days. When obvious cell cloning was observed in the culture dish, the culture medium was abandoned, and the clones were fixed with formaldehyde for 15 min, stained with gentian violet for 30 min, and the number of clones was determined under the microscope. Each experiment was repeated 3 times.

### Xenograft Tumor Model

We purchased 4-week-old B-NDG (NOD-Prkdc^scid^ Il2rg^tm1^/Bcgen) female mice from Biocytogen (Biocytogen Co., Ltd., Beijing, China) and raised them in an SPF grade environment. After 7 days of adaptive feeding, the mice were subcutaneously inoculated with HCT116-luc cells (stably expressing firefly luciferase). Ten days after inoculation, obvious xenograft formation could be seen. Ten minutes after intraperitoneal injection of D-Luciferin (3 mg/mouse), the mice were imaged, and the fluorescence values were analyzed. The mice were randomly divided into 3 groups (*n* = 3) according to the fluorescence values, and received intratumoral injection with AMO-Exos (200 μg, 50 μL), NC-Exos (200 μg, 50 μL), or PBS (50 μL) on day 0, day 3, and day 7, respectively. *In vivo* imaging was performed on the mice every 7 days. The size of the transplanted tumor was measured with vernier caliper twice a week, and the mental state and diet of the mice were observed daily.

### Statistical Analysis

Data drawing and analysis were performed using GraphPad Prism version 5.0 (GraphPad Software, San Diego, CA, United States). The data were described as mean ± standard error of the mean. The analysis of data between groups was performed using an analysis of variance or a t-test. Tumor growth in the mice was compared using Mauchly’s test of sphericity.

## Results

### Production and Identification of MSC-Derived Exosomes

**FIGURE 1:**
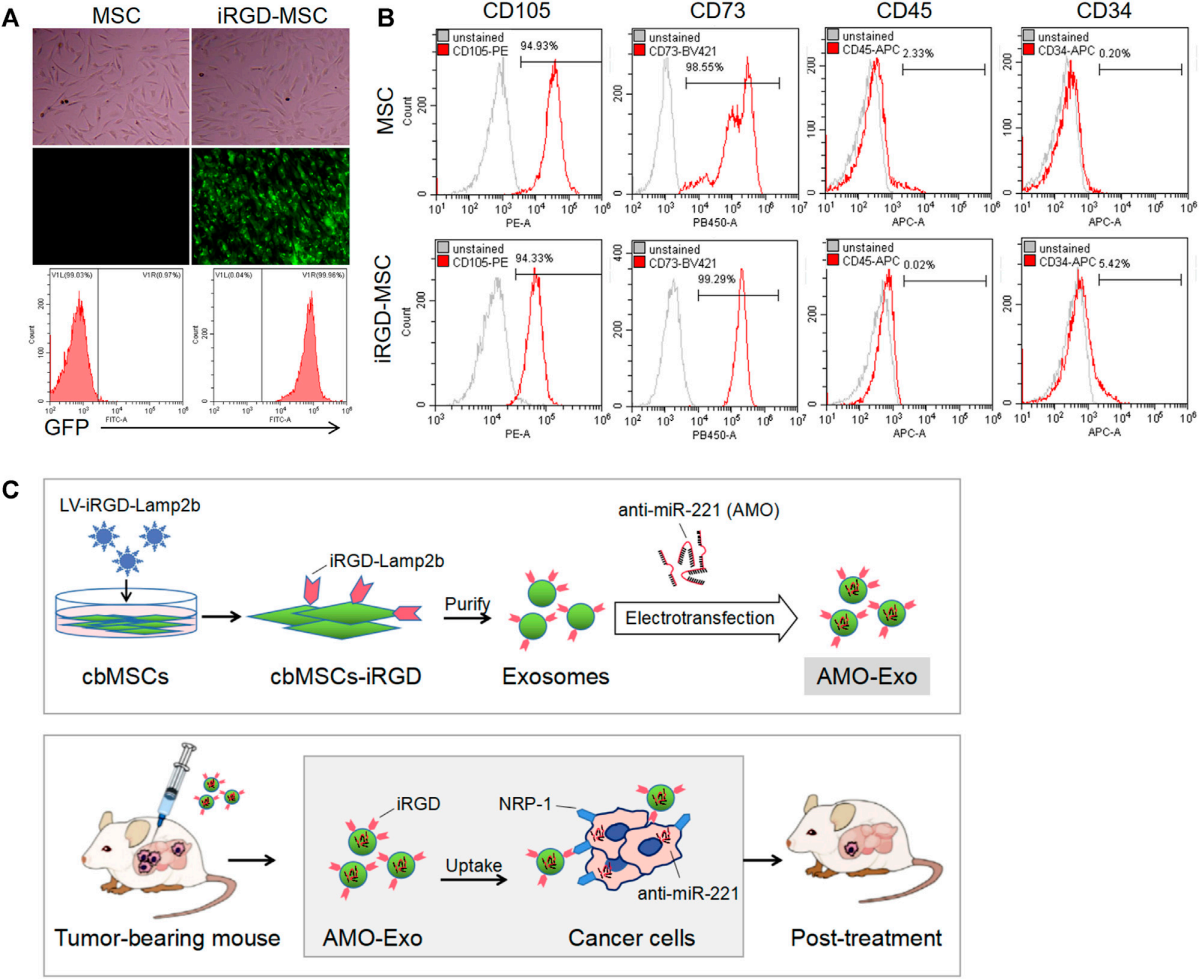
Production and identification of MSC-derived exosomes **(A)** Cell morphology and GFP expression of mesenchymal stem cells.**(B)** The expression of molecular markers on the surface of mesenchymal stem cells after iRGD peptide modification was detected by flow cytometry. **(C)** Schematic diagram of the anti-tumor mechanism of exosomal drug delivery.

We first identified the morphology and molecular markers of the cbMSCs. As shown in Figure 1A, under the light microscope, the cbMSCs were spindle-shaped and wall-attached. The flow cytometry results showed that the specific cbMSC surface markers, CD73 and CD105, were highly expressed in the cbMSCs, whereas the levels of CD34 and CD45, the specific surface markers of hematopoietic stem cells, were low (Figure 1B). In order to obtain iRGD peptide modified exosomes (iRGD-Exos), plasmids containing the iRGD-Lamp2b fusion gene were further transferred into cbMSCs using lentivirus (Figure 1C). Exosomes from iRGD-modified cbMSCs were collected and purified, and the AMOs or corresponding NCs were loaded into the exosomes by electric transduction. Subsequently, the iRGD peptide was bound to NRP-1, a receptor protein of tumor cells, to mediate the anti-tumor effect.

AMO-loaded exosomes were taken up by colon cancer cells through NRP-1.

**FIGURE 2:**
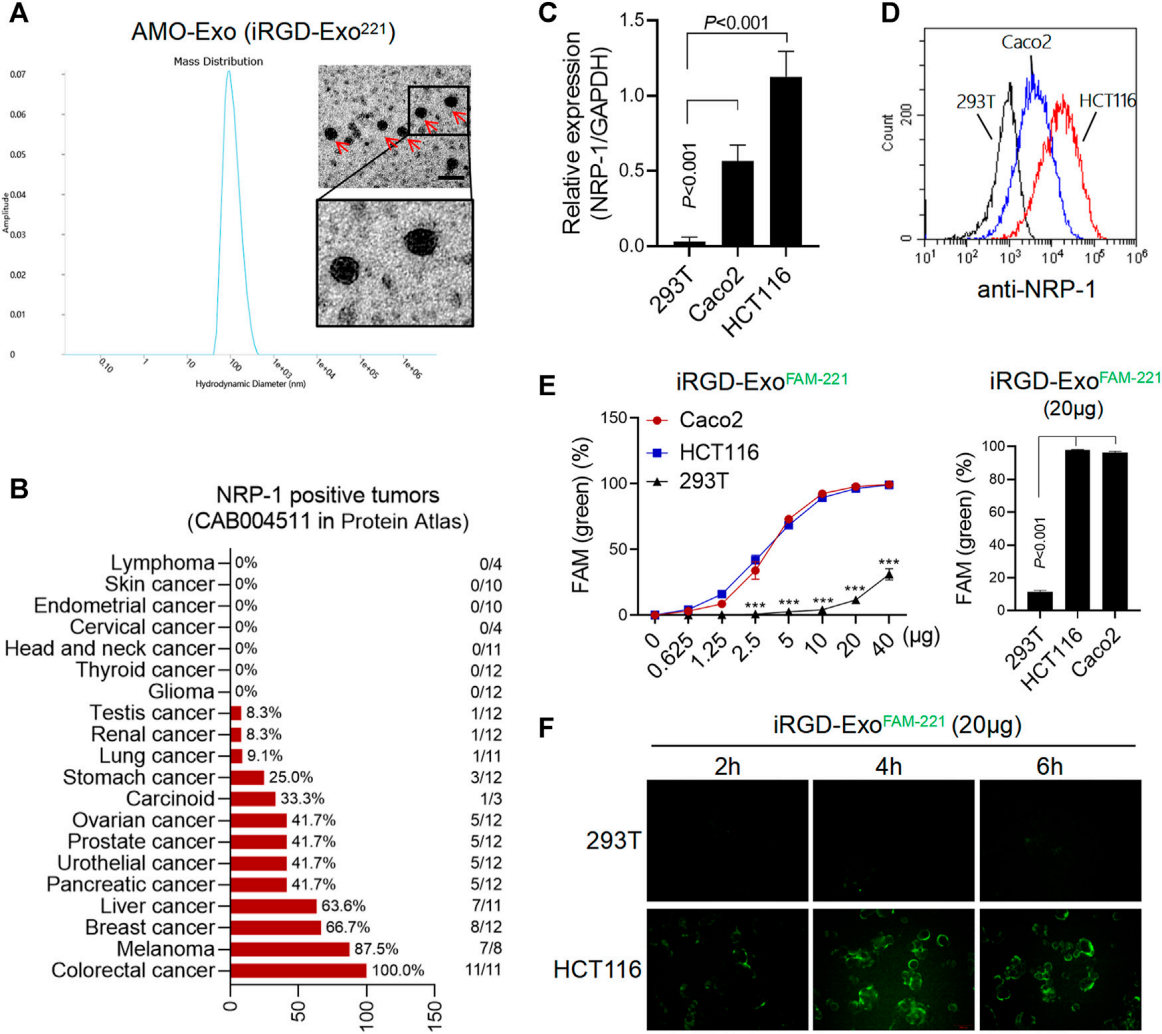
AMO-loaded exosomes were taken up by colon cancer cells through NRP-1. **(A)** Electron microscope analysis and particle size analysis of exosomes, scale bars = 200 μm. **(B)** Tissue expression of NRP-1 in a online database (The Human Protein Atlas). A majority of cancer tissues showed weak to moderate cytoplasmic immunoreactivity with a granular pattern, and the expression of NRP-1 was highest in colon cancer. **(C)** Q-PCR was used to detect the relative expression of NRP-1 in colon cancer cells (HCT116 and Caco2) and 293T cells. **(D)**The surface expression of NRP-1 in colon cancer cells was detected by flow cytometry. **(E)** After co-incubation with the FAM-labeled exosomes, the positive rate of cells expressing FAM green fluorescence was detected by flow cytometry. ***p < 0.001. **(F)** The green fluorescence signal of cells was observed by fluorescence microscopy.

The morphological identification of AMO-Exos was performed by transmission electron microscopy (Figure 2A). Considerable research has shown that the residual peptide, CRGDK/R, the product of iRGD hydrolyzation, is the specific ligand of NRP-1, which is highly expressed in tumor tissues. As shown in Figure 2B, the expression levels of NRP-1 in various tumors were analyzed. The results suggested that NRP-1 was highly expressed in malignant melanoma, breast cancer, liver cancer, and colon cancer. Quantitative PCR and flow analysis were performed, and the results showed that NRP-1 was highly expressed in the colon cancer cells Caco2 and HCT116, rather than the 293T cells (Figures 2C,D). To investigate whether iRGD-Exos could combine with NRP-1-positive colon cancer cells and deliver AMO into tumor cells, AMO was pre-labeled with fluorescein amidite (FAM) before being loaded into exosomes, and was co-cultured with human colon cancer cell lines and 293T cells. The results showed that FAM green fluorescence was increased in Caco2 and HCT116 cells in a dose-dependent manner (Figure 2E), indicating that exosomes containing FAM-AMO were taken up by colon cancer cells. Furthermore, 4 h after incubation, we observed strong green fluorescence in the HCT116 cells, but almost no fluorescence in the 293T cells (Figure 2F).

### AMO-Loaded Exosomes Inhibited Colon Cancer Cell Proliferation

**FIGURE 3:**
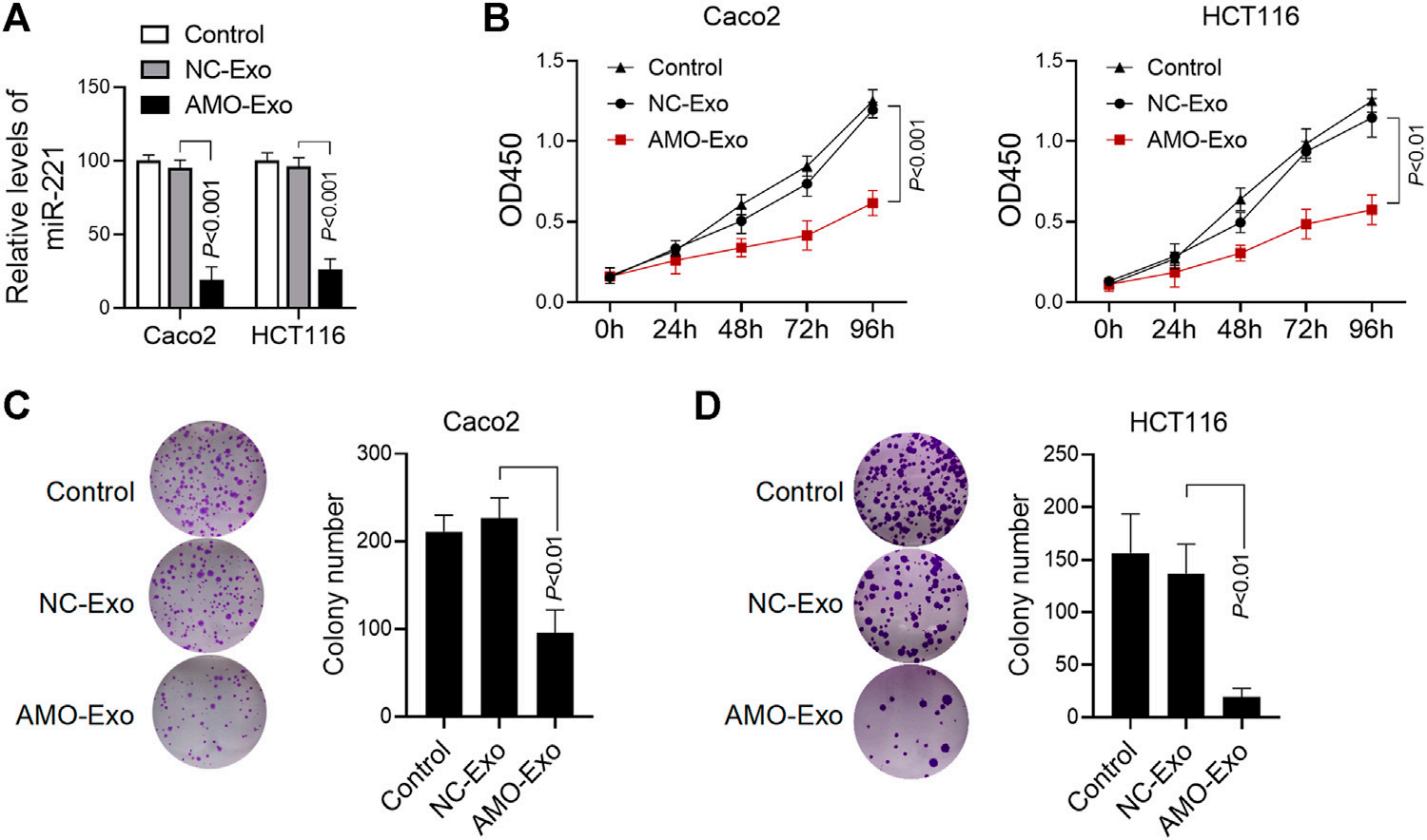
AMO-loaded exosomes inhibited colon cancer cell proliferation. **(A)** The relative expression level of miR-221 in colon cancer cells was detected by Q-PCR. Caco2 and HCT116 cells were treated with 20 μg exosomes containing anti-miRNA-221 (AMO-Exos) or their controls (AMO-NCs) for 24 h, and then lysed to detect the expression level of miR-221. **(B)** Caco2 and HCT116 cells were treated with 20 μg exosome drugs. After continuous culture for different times, the cell proliferation was evaluated. **(C)** Effect of exosome drugs on clone formation ability of Caco2 cells. Caco2 cells were treated with 20 μg exosome drugs and cultured for 14 days. The number of clones was calculated and histograms were shown. **(D)**Effect of exosome drugs on HCT116 cell clone formation ability.

We further investigated the anti-tumor function of exosomes loaded with AMO-Exos. Compared with the exosomes loaded with the NC-Exos, AMO-Exos could significantly downregulate the level of miRNA-221 in colon cancer cells Caco2 and HCT116 (Figure 3A). The results showed that AMO-Exos significantly inhibited cell proliferation, whereas no significant inhibition was observed under NC-Exo treatment (Figure 3B). Furthermore, AMO-Exos markedly suppressed the clonogenic abilities of Caco2 and HCT116 (Figures 3C,D).

### AMO-Loaded Exosomes Suppressed Tumor Growth *In Vivo*

**FIGURE 4:**
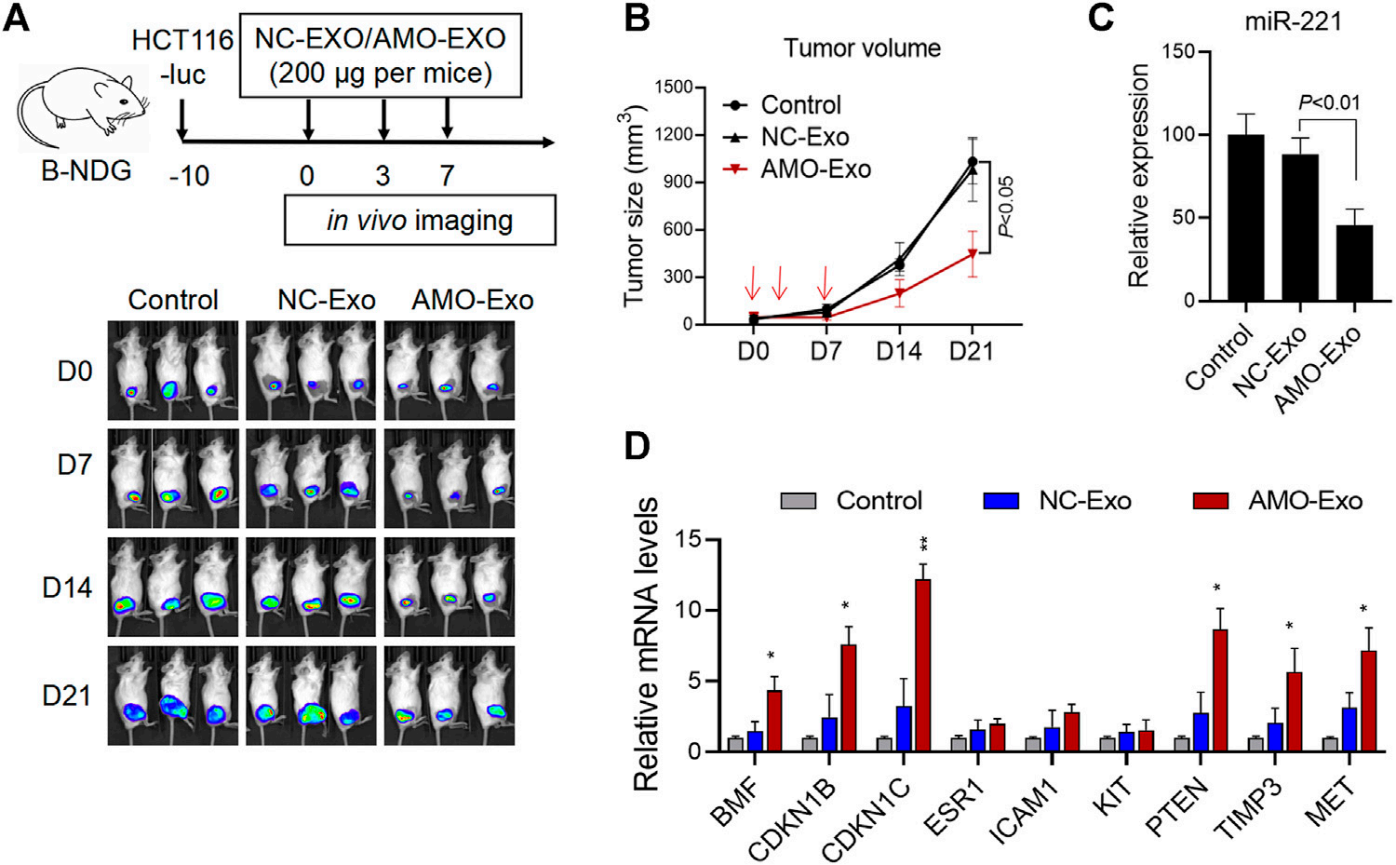
AMO-loaded exosomes suppressed tumor growth *in vivo*. **(A)** HCT116 tumor-bearing mice received 3 intratumoral injections of exosome drugs on days 0, 3, and 7. *In vivo* imaging of the mice was performed once a week to observe the change of fluorescence value. **(B)** Tumor changes in mice were measured with a vernier caliper. **(C)** The level of miR-221 in the dissected mouse tumor tissues was detected by quantitative PCR. **(D)** The mRNA levels of downstream target genes of miR-221 in the dissected mouse tumor tissues were detected by quantitative PCR. *p < 0.05, **p < 0.01.

Subcutaneous HCT116 tumor-bearing mice were established to investigate the potential anti-tumor effects of AMO-Exos *in vivo*. When the tumors formed, the mice were divided into 3 groups and received intratumoral treatment with NC-Exos or AMO-Exos 3 times (at days 0, 3, and 7). The mice were imaged *in vivo* using bioluminescent imaging every 7 days and tumor volume was measured. The results indicated that the fluorescence intensity of the mice was significantly suppressed after AMO-Exo treatment (Figure 4A), and the tumor volume also grew significantly more slowly than the control group (Figure 4B). Quantitative PCR detection of the xenograft revealed that AMO-Exo treatment significantly reduced the expression of miR-221 (Figure 4C), suggesting that anti-miR-221 transferred by exosomes acted as a tumor suppressor *in vivo*. Further analysis of the expression of downstream genes of miR-221 showed that the expression of several genes (BMF, CDKN1B, CDKN1C, PTEN, TIMP3, and MET) was significantly upregulated after AMO-Exo treatment (Figure 4D).

## Discussion

For nucleotide-based drugs, the largest obstacles to their functioning *in vivo* are the degradation of nucleases and the escape of drug molecules from the endocytes during endocytosis. In this study, cbMSC-Exos were used as micoRNA drug transport carriers. Natural exosomes lack the ability to specifically bind tumor cells because they lack a targeting property, which results in poor efficacy in tumor-targeted therapy. In our study, the iRGD-Lamp2b fusion protein was stably expressed on the surface of cbMSCs, enabling MSC-Exos to display a large number of iRGD peptides. Thus, iRGD-Exos were used as the carrier of miRNA drugs, endowing them with highly efficient tissue penetration abilities and a specific binding ability to tumor cells.

Many studies have reported that iRGD peptide accelerates drug delivery into tumor cells (Tian et al., 2014). After its combination with integrins, the enzyme hydrolysate promotes the tissue penetration of the drug (Zuo, 2019; Zhou et al., 2021). We found that NRP-1 was highly expressed in colon cancer tissues and cell lines, enabling the iRGD-Exos to be effectively ingested by colon cancer cells.

The exosomes’ lipid bilayer membrane can protect them from degradation in the blood circulation; however, this membrane structure makes it difficult for exosomes to carry “cargo.” In this study, the modified miRNA-221 was effectively loaded into exosomes by electroporation, due to easy control of the electrorevolution parameters. Wang et al. (2017) studied extracellular vesicles as a targeted delivery system for small RNA. They used electroporation to load siRNA/miRNA into the vesicles modified by nucleic acid aptamer AS1411, and then delivered siRNA/miRNA to breast cancer tissues through exosomes. Schindler et al. (Schindler et al., 2019) loaded doxorubicin into exosomes by electroporation to achieve a new drug delivery system.

The administration of exosomes is an important determinant of the efficacy and metabolic distribution of exosomes. In the HCT116 transplanted tumor mouse model, we explored local administration (intratumoral injection) and intravenous administration of exosomal drugs. The former was mainly used to observe the efficacy of the drug and the latter to assess its metabolism. Our results showed that 3 intratumoral injections inhibited the growth of the transplanted tumor to a certain extent; however, the tumor eventually recurred. Increasing the dose and frequency of administration will be further studied. Preliminary experiments have also been done on the distribution of exosomes, and exosomes were observed in the liver, spleen, and lung through *in vivo* imaging (data not shown), which was consistent with previous studies (Yi et al., 2020). The toxicity and duration of exosome drugs *in vivo* should be explored in subsequent studies.

Previous studies have confirmed that miR-221 also inhibits the expressions of the PTEN and cyclin-dependent kinase inhibitor family members (Le Sage et al., 2007; Park et al., 2011; Callegari et al., 2012), whereas anti-miR-221 upregulates the expression levels of corresponding tumor suppressor factors, which could be the molecular mechanism underlying the anti-tumor effect induced by anti-miR-221 in this study. This finding is consistent with the latest understanding of miRNA, i.e., that miRNA and its target mRNA are not in a simple “one-to-one” direct linear relationship, but constitute a complex network regulation pattern (Liu et al., 2018).

## Conclusion

In this study, we described a novel drug delivery system that infiltrated anti-miRNA-221 into solid tumors using cbMSC-derived exosomes. The modified exosomes showed high binding ability to NRP-1-positive colon cancer cells and significantly inhibited tumor growth *in vitro* and *in vivo*. Our data suggest that iRGD-modified cbMSCs-derived exosomes appear to be one of the best candidates for the specific transport of miRNA drugs to tumors.

## Data Availability

The original contributions presented in the study are included in the article/supplementary material, further inquiries can be directed to the corresponding authors.
